# Giant renormalization of dopant impurity levels in 2D semiconductor MoS_2_

**DOI:** 10.1038/s41598-020-61675-y

**Published:** 2020-03-18

**Authors:** Jeongwoon Hwang, Chenxi Zhang, Yong-Sung Kim, Robert M. Wallace, Kyeongjae Cho

**Affiliations:** 1 Department of Materials Science and Engineering, University of Texas at Dallas, Richardson, Texas 75080 USA; 2 Present Address: Department of Physics Education, Chonnam National University, Gwangju, 61186 Korea; 3 Korea Research Institute of Standards and Science, Yuseong, Daejeon 305-340 Korea

**Keywords:** Materials science, Nanoscience and technology

## Abstract

Substitutional doping in 2D semiconductor MoS_2_ was investigated by charge transition level (CTL) calculations for Nitrogen group (N, P, As, Sb) and Halogen group (F, Cl, Br, I) dopants at the S site of monolayer MoS_2_. Both n-type and p-type dopant levels are calculated to be deep mid-gap states (~1 eV from band edges) from DFT total energy-based CTL and separate DFT + GW calculations. The deep dopant levels result from the giant renormalization of hydrogen-like defect states by reduced dielectric screening in ultrathin 2D films. Theoretical analysis based on Keldysh formulation provides a consistent impurity binding energy of ~1 eV for dielectric thin films. These findings of intrinsic deep impurity levels in 2D semiconductors MoS_2_ may be applicable to diverse novel emerging device applications.

## Introduction

As the conventional microelectronic device scaling is rapidly approaching the physical limits of 7–13 nm nodes based on EUV technology within 10 years^1^, the research community has been searching for nanoscale materials platforms as well as alternative device technologies such as multi-level^2,3^, neuromorphic^4,5^, or quantum computing devices^6^. To meet the increasing requirements of scaled device configurations, nanoscale materials (such as carbon nanotubes, semiconductor nanowires, graphene and 2D semiconductors) have been extensively investigated over the last two decades as an alternative material platform to continue the device scaling of silicon-based microelectronics technology. 2D semiconductors, such as MoS_2_ and other transition metal dichalcogenides (TMDs), have been most recently investigated, but there are many practical challenges in addition to their promising device materials characteristics. One of the challenges has been effective doping of 2D semiconductors which are known to be very difficult without clear understanding on the underlying reasons for such difficulty. Previous modeling studies based on density functional theory (DFT) calculations have investigated the possibility of chalcogen substitutional doping for monolayer TMDs^7,8^, which is a seemingly promising approach to induce p-type or n-type doping based on the well-established doping method in 3D semiconductors (e.g., B or P substitution in Si). In both DFT studies, the energy difference between impurity level and conduction band edge (valence band edge) was interpreted as the binding energies of the electron (hole) for n-type (p-type) dopants. Based on the interpretation, Cl_S_ and Br_S_ (P_S_ and As_S_) dopants were identified as promising n-type (p-type) dopants in MoS_2_ (subscript s represents S substitution). However, it is important to note that the *Kohn-Sham (KS) eigenvalues of the impurity levels are not quasiparticle energies*, and therefore, the binding energy, which is the excitation energy of bound charge carriers to the band edges, cannot be directly calculated from the difference between KS eigenvalues as indicated in the previous studies. Instead, it should be determined through the total energy difference between different charge states of the impurity^9^, which is the well-established charge transition level (CTL) analysis to calculate impurity levels in bulk semiconductors with quantitative experimental validations. Recently, the CTL analysis has been applied to investigate the effects of native defects and metal-site substitution for 2D TMDs providing a good agreement with experimental results^10–12^. Thus, we have systematically examined the chalcogen-site dopant levels in a prototype 2D semiconductor MoS_2_ based on CTL analysis by employing accurate DFT and GW calculation methods and elucidated the underlying mechanism of deep dopant level formation in 2D semiconductors.

In this work, we investigate the impurity states in monolayer MoS_2_ induced via the substitution of S by Nitrogen group (N, P, As, Sb) and by Halogen group (F, Cl, Br, I) elements. The impurity levels are calculated based on the CTL analysis with the correction on total energy for charged defects^11^. For N-substituted case, we employ the DFT + GW formalism to crosscheck the validity of our CTL results based on total energy methods, and those two approaches for CTL calculation give a consistent result. The predicted deep impurity levels (~1 eV) are attributed to the reduced screening in 2D materials leading to a giant renormalization of impurity levels. Our results indicate that the impurity bound state induced in free-standing 2D TMDs through chalcogen substitution by nitrogen or halogen group elements are robust from thermal excitation at room temperature. This finding suggests that alternative doping strategies are required to enable controlled n-type and p-type doping of 2D semiconductors.

## Results and Discussion

**Figure 1:**
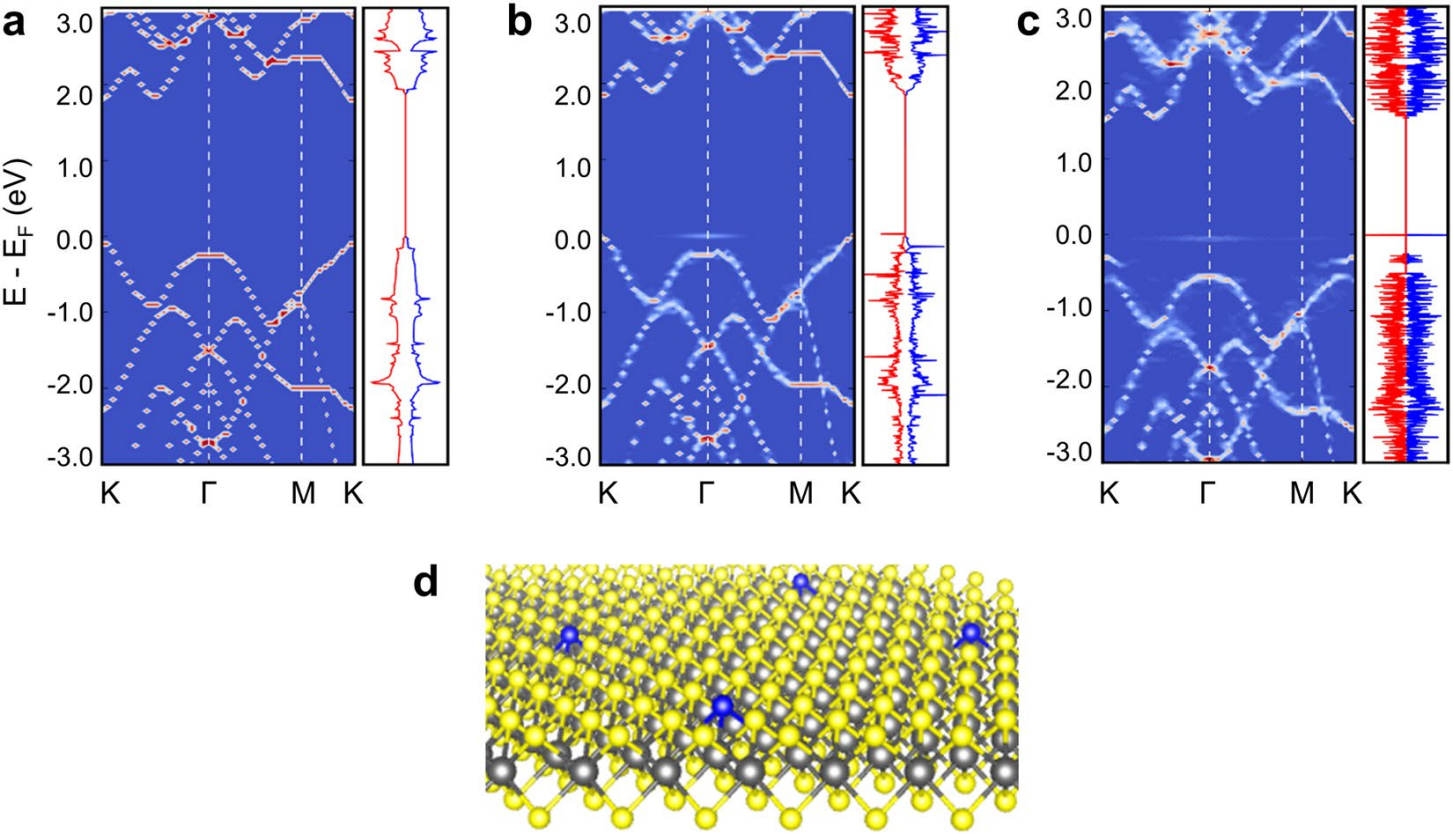
Electronic structures of MoS_2_ with and without S-substitution by P and atomic structure of P-substituted MoS_2_. The band structures, which are unfolded into the first Brillouin zone of the primitive cell, and corresponding DOS (red for spin up and blue for spin down) of (**a**) pristine monolayer MoS_2,_ (**b**) P-substituted monolayer MoS_2_ at charge state of q = 0, and (**c**) P-substituted monolayer MoS_2_ at charge state of q = −1. (**d**) The atomic structure of P-substituted monolayer MoS_2_ is shown, where one S atom (yellow ball) in a 6 × 6 supercell of MoS_2_ is replaced by one P atom (blue ball)^37^.

First, we investigate the effect of chalcogen site substitution on the electronic structure of monolayer MoS_2_ with one chalcogen atom replaced by group-V and group-VII elements in a 6 × 6 supercell. The band structure and density of states (DOS) of pristine MoS_2_ are shown in Fig. 1a, in which the 6 × 6 supercell band structure is unfolded to the first Brillouin zone of the primitive cell. The substitution of one S atom by a P atom in the 6×6 supercell perturbs the band structure of host MoS_2_ by introducing an acceptor level just above the valance band edge as shown in Fig. 1b. The induced in-gap state near the valence band edge is also clearly shown as a sharp peak in the corresponding DOS. This impurity level analysis based on the KS eigenvalues in DFT calculations is consistent with previous DFT studies of P_S_ dopant levels in MoS_2_, which was interpreted as a promising p-type dopant candidate^7,8^. However, even within the scheme of interpreting the KS eigenvalue as the impurity level, there is an uncertainty in choosing the KS eigenvalues between neutral and charged systems. When the impurity state is doubly occupied (i.e., q = −1 state), the impurity level in the band structure or DOS is shifted deeper into the band gap as shown in Fig. 1c. This KS eigenvalue shift is attributed to the charging effect caused by the Coulomb repulsion between the spin up and spin down electrons sitting on the localized impurity site. Regardless of the charging effect on KS eigenvalues, there are much more significant many-body effects missing in such analysis, and it would be misleading to identify the results in Fig. 1 as a shallow P_S_ acceptor level.

**Figure 2:**
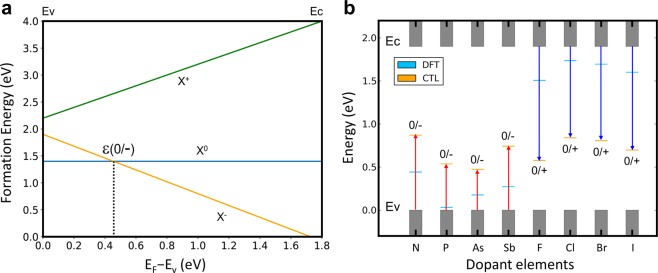
Charge transition levels (CTLs) based on DFT total energy methods. (**a**) Illustrative description of CTL. Formation energies of impurity (X) at different charge states, i.e. +1, 0, and −1, are shown as a function of Fermi level (i.e., electron chemical potential). The Fermi level at the crossing point of the two lines designated by X° (blue) and X^−^ (orange) is the CTL ε(0/−) from neutral state to negatively charged state. (**b**) Calculated CTLs based on the DFT total energy method are indicated by orange bars with the mark 0/+ or 0/−. For clear comparison, KS eigenvalues of impurity states are also shown (blue bars). Valence and conduction bands are represented by gray shaded columns. Here, valence band maximum is set to be the reference energy. The red (blue) arrows indicates how much the Fermi level should change with respect to valence band maximum (conduction band minimum) to induce the charge state transition of the impurities.

The many-body effects in impurity level calculations can be included in the accurate CTL methods based on the DFT total energy calculations of charged systems or GW calculations combined with DFT calculations (DFT + GW). We first apply the DFT total energy calculations of charged systems to predict the CTLs of the selected p-type and n-type dopant candidates in MoS_2_. Since additional charge is localized at the impurity site, we employ charge correction method of Noh *et al*.^11^ with a Gaussian model charge to remove spurious long-range coulomb interaction between charged impurities due to imposed periodic boundary conditions (see Supplementary for details). Subsequently, we apply DFT + GW method to N_S_ dopant and compare with the CTL calculated from DFT total energy method for an additional validation of the predicted CTL values. The CTL is defined as the value of the electron chemical potential ε(q/q − 1) at which the charge state of the impurity changes from q to q–1 as illustrated in Fig. 2a, where the formation energies of the system with an impurity (X) at different charge states are calculated by using the Eq. (1). The formation energy of charged impurity is linearly dependent on the Fermi level, while that of neutral system remains constant. The Fermi level at the crossing point of the two lines designated by X^0^ and X^−^ is the CTL from q = 0 state to q = −1 state, ε(0/−1). If the Fermi level is increased above this level, the impurity with q = −1 becomes energetically favorable.

We applied this method to all the group-V and group-VII dopant elements, which are summarized as the calculated CTLs in Fig. 2b. We note that for the group-V dopants, the Fermi level should be above the VBM by the CTL value to introduce the charge transition (0/−1). Conversely, in the case of group-VII dopants, it should be interpreted that the Fermi level should be below the CBM by (E_gap_ – CTL value) to introduce the charge transition (0/+1). The exact CTL values are given in Supplementary Table 1. All the group-V and group-VII elements are found to introduce deep impurity levels that can hardly donate (accept) charges to (from) conduction (valance) band of the host MoS_2_. For comparison, the positions of KS eigenstates with respect to valence band maximum are also shown, based on which chalcogen substitution by Group-V elements or group-VII elements is expected to introduce p-type and n-type doping, respectively. (See Supplementary Figs. 1 and 2 for the respective density of states.) Specifically, the KS eigenstates indicate that among the group-V elements, P_S_ and As_S_ dopants seem promising to induce the p-type doping agreeing with previous DFT studies^7,8^. This discrepancy between the CTLs and KS eigenstates originates from the limited nature of KS eigenvalues which do not fully account the many-body effects, and its limitation becomes more significant in low-dimensional systems such as 2D materials with reduced screening. Our results clearly show that interpreting the energy difference between KS levels as excitation energy of electron or hole is a misconception without including the impurity level renormalization energy contribution. These deep impurity levels with large binding energies are consistent with the predicted binding energy of impurity state in an ultrathin dielectric film. Specifically, when we employ the Keldysh formulation^13^ (since we are considering free-standing monolayer MoS_2_, the original Keldysh formulation now turns into ${E}_{b} \sim \frac{{e}^{2}}{\varepsilon d}\,\mathrm{ln}\left[{\left(\frac{\varepsilon }{{\varepsilon }_{0}}\right)}^{2}\frac{d}{{a}_{0}}\right]$, where *a*_0_ is the effective Bohr radius of electron in bulk MoS_2_) with the in-plane dielectric constant ε = 15ε_0_, effective thickness ~6 Å^11^, and effective mass m* ~0.4 m_e_^14^ of monolayer MoS_2_, the resulting impurity state binding energy becomes ~1 eV for the thin film compared to 24 meV for bulk, consistent with the calculated CTL values. These results suggest that dopants will produce deep impurity levels, and that other approaches such as surface charge transfer through Li adsorption^15^ would be more practical to induce doping in 2D semiconductors.

**Figure 3:**
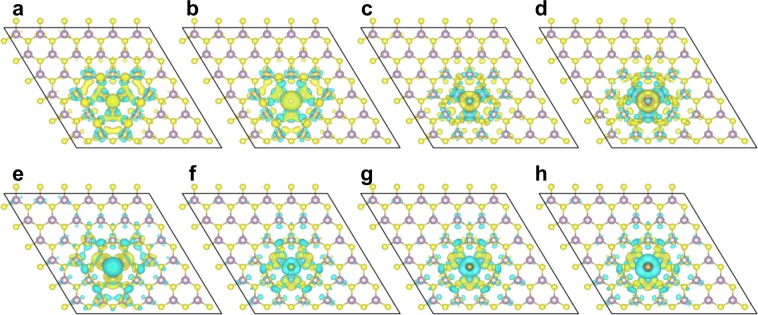
Isosurface of charge-density difference between negatively (or positively) charged system and neutral system. (**a**) N-MoS_2_. (**b**) P- MoS_2_. (**c**) As-MoS_2_. (**d**) Sb-MoS_2_. (**e**) F-MoS_2_. (**f**) Cl-MoS_2_. (**g**) Br-MoS_2_. (**h**) I- MoS_2_. Yellow cloud indicates the regions of charge gain, and blue cloud indicates the regions of charge loss^37^.

We note that the calculated CTLs of dopant elements in the same group vary up to 0.4 eV. Although the impurity atoms are in the same group (either group-V or group-VII) and have the same number of valence electrons, the hydrogen-like defect model does not include all the electronic screening effects of the impurity atoms. Specifically, their atomic radii and the local structure of optimized geometries are different. For instance, the impurity atom with larger atomic radius compared to that of S protrudes out of the MoS_2_ plane (see Table 1). Furthermore, different impurity atoms have different valence electronic shell size and polarizability leading to different local screening at the impurity site. It is worthwhile to note similar (but much smaller due to 1/ε^2^ bulk dielectric screening) dopant level differences for donor and acceptor in bulk Si. Consequently, the different bonding geometry and different electronic polarizability of each element affect the charge transition level much more than bulk semiconductors. We note that while the impurity element with larger distance from Mo plane tends to have larger CTL value, the smallest element (i.e. N and F, for each group), of which the distance from the Mo plane is much shorter than the distance between Mo plane and S plane in pristine MoS_2_, has the largest CTL value. This result is tabulated in Table 1. Also, the charge-density difference plots of investigated systems are shown in Fig. 3.

**Table 1:** Local atomic geometry around the impurity atom.

Element X	N	P	As	Sb	F	Cl	Br	I	Pristine MoS_2_
d_x-Mo_ (Å)	0.90	1.53	1.80	2.07	1.29	1.68	1.91	2.19	1.56
d_Mo-S_ (Å)	1.77	1.55	1.56	1.54	1.63	1.51	1.45	1.39	1.56
d_X-S_ (Å)	2.67	3.07	3.36	3.61	2.92	3.20	3.36	3.58	3.11

d_x-Mo_ indicates the distance between impurity element X (X = N, P, As, Sb, F, Cl, Br, I, and S for pristine MoS_2_) and Mo plane. d_Mo-S_ indicates the distance between Mo plane and S atom on the opposite side of the impurity element X. d_X-S_ (Å) is the sum of d_x-Mo_ and d_Mo-S_.

Another quantitative observation in Fig. 2b is that CTLs of group-VII elements (the relative position from CBM) are generally deeper than those of group-V elements (the relative position from VBM) with correspondingly larger impurity level renormalization energy. In the hydrogen-like model for 3D semiconductor or the modified version for ultratin film, the binding energy is determined by the macroscopic dielectric constant and effective mass of the host material without including local electronic structure effect at imputity sites, leading to symmetric n-type and p-type level positions from the band edges. However, our CTL calculation results show different trends between n-type and p-type impurity binding energies indicating different impurity level renormalization energies. This difference is atrributed to the smaller local plorizability of the group-VII impurities in the charge state of q = +1, i.e. cation, compared to the group-V impurities in the charge state of q = −1, i.e. anion^16^. At large distance from the impurity, the screening of Coulomb interaction is governed by the macroscopic dielectric constant of the host material, i.e. MoS_2_. However, near the localized impurity site, the local polarizabity plays a central role^17^. The poor local polarizability of cationic impurities cannot effectively screen the Coulomb interaction between the impurity nucleus and electron when one electron is bound, i.e. transition from q = +1 to q = 0, which results in stronger Coulomb interaction with larger binding energies.

**Figure 4:**
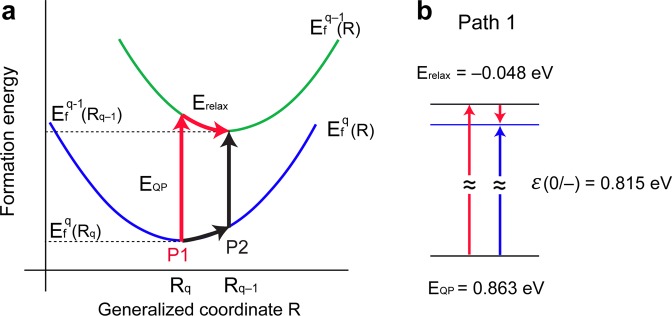
CTL based on combined DFT and GW formalism. (**a**) Schematic description for calculating CTL in the combined DFT and GW formalism, where E_relax_ is obatined from DFT total energy calculations, and E_QP_ is calculated from GW calculation. The formation energy is a state function, so that the CTL values calcualted following path 1 (P1) or path 2 (P2) result in the same ε(q/q^−^1). (**b**) Calculated CTL of N-substituted MoS_2_ from neutral state to negatively charged state as following P1 shown in **a**.

As an alternative approach for calculating impurity levels, we employ the combined DFT and GW formalism to crosscheck the accuracy of our CTL results based on DFT total energy calculations. N-substituted MoS_2_ is chosen as the prototype system for comparison. First, we confirm that our G_0_W_0_ calculation for pristine MoS_2_ gives a direct band gap of 2.80 eV at K point. The atomic configuration of neutral system is used to perform GW calculation of q = −1 system, i.e. one more electron, and quasiparticle excitation energy is determined from the ionization potential along path P1 shown in Fig. 4a. As shown in Eq. (3), ε(q/q − 1) can be divided into two contibutions, i.e. the structural relaxation energy and the electronic excitation energy, and the calculated results are summarized in Fig. 4b. E_QP_ includes the aforementioned electrostatic correction, which pushes up the negatively charged impurity level from the VBM, consistent with the previous studies for point defects in LiF, SiO_2_ and SiC^9^. The resulting impurity level of 0.815 eV from DFT + GW calculations agrees well with our result of N_S_ CTL of 0.865 eV as shown in Fig. 2b.

**Figure 5:**
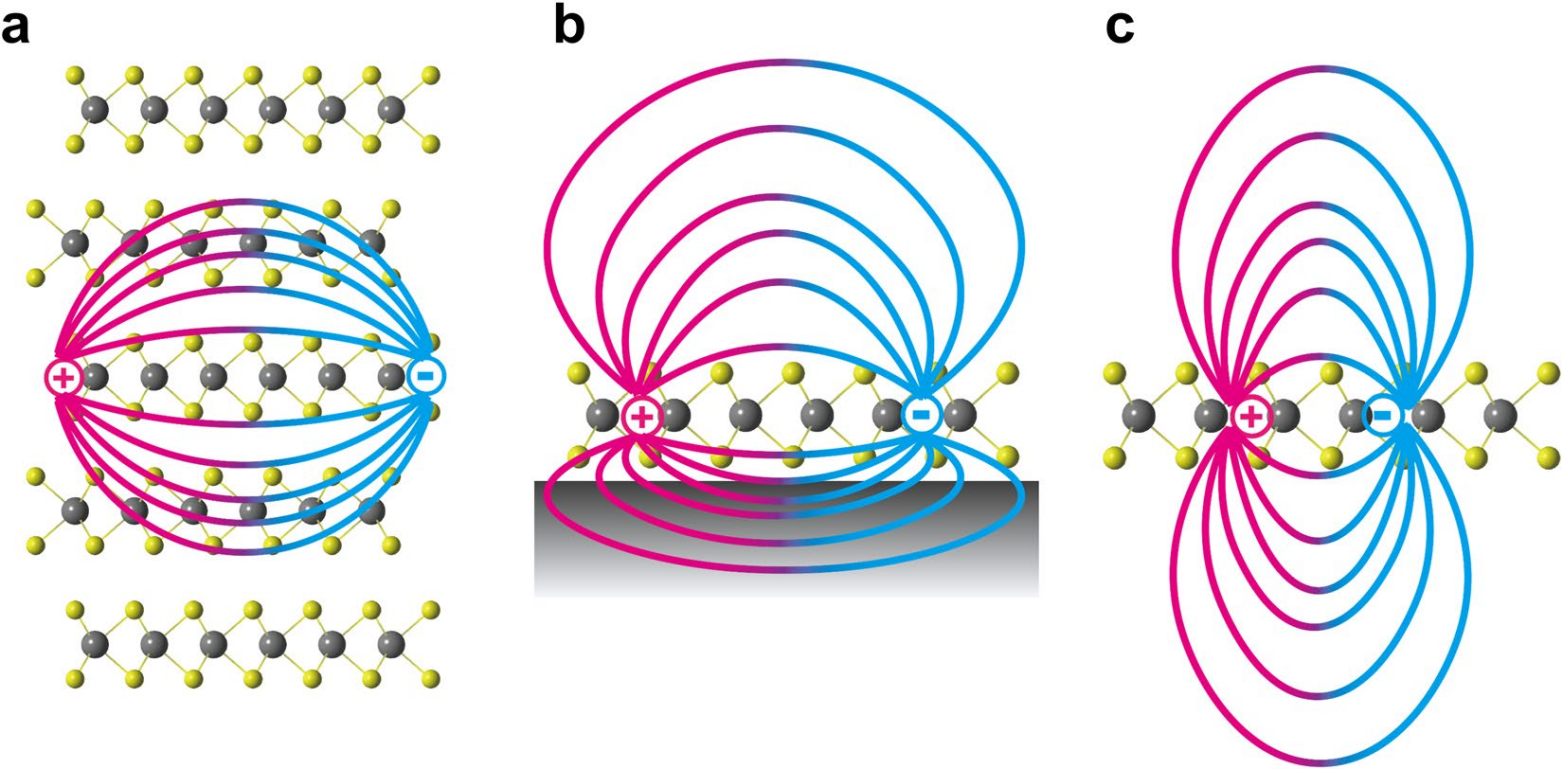
Schematic illustration of the dielectric screening of Coulomb interaction. Due to the ultrathin body of 2D materials, the Coulomb interaction between charged particles inside the system is sensitive to the environment as represented by the relative distances between charged particles and different ranges of electric field lines in the (**a**) multilayer MoS_2_, (**b**) monolayer MoS_2_ on a dielectric substrate, and (**c**) free-standing monolayer MoS_2_^23^. As illustrated, the Coulomb interaction within 2D materials can be modified through environmental dielectric screening.

Finally, it is worthwhile to note that the current analysis is rigorously applicable to an isolated monolayer MoS_2_, and that the impurity level analysis for multilayer systems and monolayer on substrate or dielectric encapsulation would require separate modeling analysis of CTLs by including the environment effects on the impurity level renormalization energy. We note that some careful clarification is required when comparing the results of theoretical and experimental works since experiments are usually carried out for few layer TMDs^18–22^ in the vertically stacked configuration on dielectric substrate. The effects of dielectric screening within multilayers, which was well-illustrated by Chernikov *et al*. for exciton states^23^, and from substrates are expected to reduce the binding energy of the impurity states considerably as illustrated in Fig. 5a–c. Applying the previously used Keldysh formulation, we can estimate that the impurity state binding energy will decrease as ~1/*d* for multilayer TMDs and similarly to substrate dielectric effects. For the multilayer TMDs, the van der Waals stack of doped and intrinsic TMD monolayers (Fig. 5a) further complicates the local chemical potential change of doped layer from the intrinsic layer leading to realignment of 2D band structure. A systematic analysis of dopant levels in multilayer TMD systems will require separate modeling studies based on CTL calculations. For a monolayer TMD, the environmental dielectric effect is shown to have a strong influence on the electronic structure^24^. By replacing the vacuum environment of doped monolayer TMD within the Keldysh formulation, the impurity level depends on the top and bottom dielectric constants (*ε*_1_ and *ε*_2_) as $\sim \,\mathrm{ln}\left[{\left(\frac{2\varepsilon }{{\varepsilon }_{1}+{\varepsilon }_{2}}\right)}^{2}\frac{d}{{a}_{0}}\right]$ leading to shallow impurity levels for increasing dielectric screening of the environment^13^. Our CTL prediction and giant renormalization of dopant impurity levels are consistent with previous studies on the renormalization of band gap and exciton binding energy in 2D materials due to the environmental dielectric effect^24–26^. This finding on the intrinsic nature of deep dopant levels suggests that a conventional doping strategy developed for bulk semiconductors is not directly applicable to 2D semiconductors, and that a novel doping strategy (such as ionic gating) is required to effectively dope 2D semiconductors.

## Conclusions

We have investigated the possibility of substitutional doping of monolayer MoS_2_ by introducing the group-V and group-VII elements at the chalcogen site. The calculated CTLs of impurities are found to be deep levels (~1 eV), which is consistent with the theoretical analysis based on Keldysh formulation. These impurities with deep CTLs would not function as active charge sources by thermal excitation for the host material at room temperature. The large renormalization of impurity state binding energy is the consequence of a reduced dielectric screening in monolayer MoS_2_, which is a general property of 2D semiconductors, but not present in the conventional 3D semiconductors. Furthermore, due to the ultra-thin thickness and large surface area, the electronic properties of the 2D materials can be readily modified by the environmental effects, and experimental confirmation of the predicted impurity level renormalization would require careful setup to minimize the spurious environmental screening effects. Once the predicted CTLs are firmly established by experimental validations, there would be many possible strategies to engineer environmental screening leading to controlled impurity level renormalization and dopant level activation for novel 2D semiconductor device applications.

## Methods

We performed DFT calculations with local density approximation (LDA) for exchange-correlation functional^27^ using projector-augmented wave (PAW) pseudopotentials^28,29^ implemented in the Vienna ab initio simulation package^30,31^. The wave functions were expanded in plane waves with an energy cutoff of 450 eV. The uniform k-point grid of 6 × 6 × 1 (3 × 3 × 1) was used for electronic (geometry) optimization for a 6 × 6 supercell including 108 atoms. Atomic coordinates were relaxed until the force on each atom was less than 0.01 eV/Å. To analyze the effect of impurity level on the band structure of monolayer MoS_2_, the supercell band structure was unfolded to the first Brillouin zone of the primitive cell by employing the method developed by Medeiros *et al*.^32^.

The formation energies of a dopant impurity at different charge states were calculated with the following formula:

1 $${{\rm{E}}}_{{\rm{f}}}^{{\rm{q}}}={{\rm{E}}}_{{\rm{tot}}}^{{\rm{defect}}}({\rm{q}})-{{\rm{E}}}_{{\rm{tot}}}^{{\rm{pristine}}}-{\sum }^{}{{\rm{N}}}_{{\rm{i}}}{{\rm{\mu }}}_{{\rm{i}}}+{\rm{q}}({{\rm{\varepsilon }}}_{{\rm{v}}}+{{\rm{\varepsilon }}}_{{\rm{F}}})+{{\rm{E}}}_{{\rm{corr}}},$$

where ${{\rm{E}}}_{{\rm{tot}}}^{{\rm{pristine}}}$ is the total energy of a supercell of pristine MoS_2_ without a defect, E_tot_ is the total energy of MoS_2_ containing an impurity in the supercell, N_i_ is the number of element i (Mo, S, or impurity atom) added (or removed with a minus sign) in the supercell, μ_i_ is the chemical potential of the element i, q is the charge state of the defect, ε_v_ is the valence band maximum (VBM) level of the pristine MoS_2_, ε_F_ is the Fermi level with reference to the ε_v_, and E_corr_ is the total energy correction including the level alignment and removing spurious electrostatic interaction between image charges. For a periodic cell with a charged defect, the spurious long-range Coulomb interaction due to the repeated images and neutralizing background charge introduces errors in the total energy, which should be removed^11^. The charge correction method is described in the Supplementary information in details.

The CTL can be written in terms of formation energies as

2 $${\rm{\varepsilon }}({\rm{q}}/{\rm{q}}-1)=[{{\rm{E}}}_{{\rm{f}}}^{{\rm{q}}-1}-{{\rm{E}}}_{{\rm{f}}}^{{\rm{q}}}]{|}_{{{\rm{\mu }}}_{{\rm{el}}}={{\rm{\varepsilon }}}_{{\rm{v}}.}}$$

As an alternative approach, we also employed combined DFT and GW formalism^33,34^, where CTL can be separated into structural relaxation energy (E_relax_) and electronic excitation energy (E_QP_);

3 $$\begin{array}{rcl}{\rm{\varepsilon }}({\rm{q}}/{\rm{q}}-1) & = & {{\rm{E}}}_{{\rm{f}}}^{{\rm{q}}-1}({{\rm{R}}}_{{\rm{q}}-1})-{{\rm{E}}}_{{\rm{f}}}^{{\rm{q}}}({{\rm{R}}}_{{\rm{q}}})\\  & = & [{{\rm{E}}}_{{\rm{f}}}^{{\rm{q}}-1}({{\rm{R}}}_{{\rm{q}}-1})-{{\rm{E}}}_{{\rm{f}}}^{{\rm{q}}-1}({{\rm{R}}}_{{\rm{q}}})]+[{{\rm{E}}}_{{\rm{f}}}^{{\rm{q}}-1}({{\rm{R}}}_{{\rm{q}}})-{{\rm{E}}}_{{\rm{f}}}^{{\rm{q}}}({{\rm{R}}}_{{\rm{q}}})]\\  & = & {{\rm{E}}}_{{\rm{relax}}}+{{\rm{E}}}_{{\rm{QP}}},\end{array}$$

where R_q_ is the equilibrium configuration of the charge state q. E_relax_ and E_QP_ were obtained from DFT total energy calculation and GW calculation, respectively. Equivalently, ${{\rm{E}}}_{{\rm{f}}}^{{\rm{q}}}({{\rm{R}}}_{{\rm{q}}-1})$ can be added and subtracted to give different values of E_relax_ and E_QP_, which results in the same ${\rm{\varepsilon }}({\rm{q}}/{\rm{q}}-1)$. We calculated quasiparticle energies within the G_0_W_0_ approximation with the BERKELEYGW package^35^ for 3 × 3 supercell including 27 atoms. The dielectric matrix and self-energy were calculated with a truncated Coulomb interaction^36^ on a 4 × 4 × 1 k-point grid with a 6 Ry energy cutoff. Electrostatic correction on the quasiparticle energy was made following the method proposed by Jain *et al*.^34^.

## Supplementary information

Supplementary Information.
