# Supplementary material for: Giant renormalization of dopant impurity levels in 2D semiconductor MoS2
Source: Sci Rep. 2020 Mar 18;10:4938. doi: 10.1038/s41598-020-61675-y (PMC7080777; doi:10.1038/s41598-020-61675-y)
Supplement: Supplementary file 1 — Supplementary Information. [file 41598_2020_61675_MOESM1_ESM.pdf]

Supplementary Information

Giant renormalization of dopant impurity levels in 2D semiconductor MoS_2

Jeongwoon Hwang[1,2], Chenxi Zhang[1], Yong-Sung Kim[3], Robert M. Wallace[1], and Kyeongjae Cho[1]*

[1]Department of Materials Science and Engineering, University of Texas at Dallas, Richardson, Texas 75080, USA

[2]Current address: Department of Physics Education, Chonnam National University, Gwangju 61186, Korea

[3]Korea Research Institute of Standards and Science, Yuseong, Daejeon 305-340, Korea

*Corresponding author: kjcho@utdallas.edu

Electronic structure of MoS_2 with dopant impurity

We perform the electronic structure analysis and investigate the effect of the impurity dopants on the density of states (DOS) of monolayer MoS_2. In addition to the neutral case, we also examine the electronic structure of MoS_2 with dopant impurity at a different charge state (i.e. q= +1 or q= -1). In Fig. S1, the dopant-induced Kohn-Sham (KS) states appear close to the valence band maximum (VBM), and therefore substitution of group-V elements at S site seems promising to introduce p-type doping. Conversely, the substitutional doping of group-VII elements at S site (see Fig. S2), introduces KS states close to the conduction band minimum (CBM). However, as emphasized in the main text, the KS eigenvalues of the impurity levels are not quasiparticle energies and should not be misunderstood as excitation energies or binding energies.

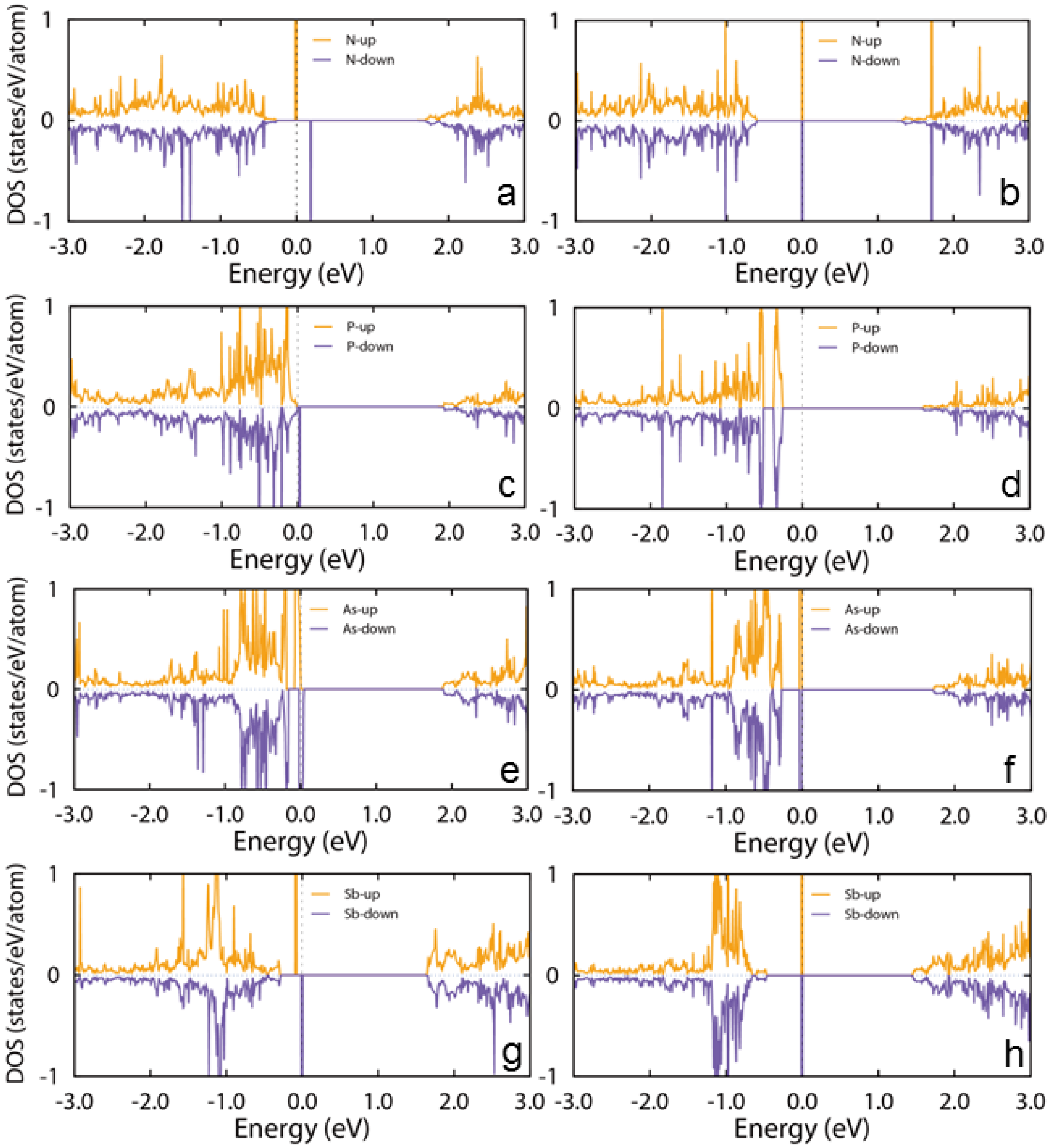

Figure S1. The density of states (DOS) of the substitutionally doped monolayer MoS_2 with Group-V elements. The spin up and spin down states are shown as orange and purple solid lines, respectively. The left column is the DOS of (a) N-doped (c) P-doped (e) As-doped (g) Sb-doped monolayer MoS_2 at neutral charge state (q=0), and the right column is the DOS of (b) N-doped (d) P-doped (f) As-doped (h) Sb-doped monolayer MoS_2 at negatively charged state (q= -1).

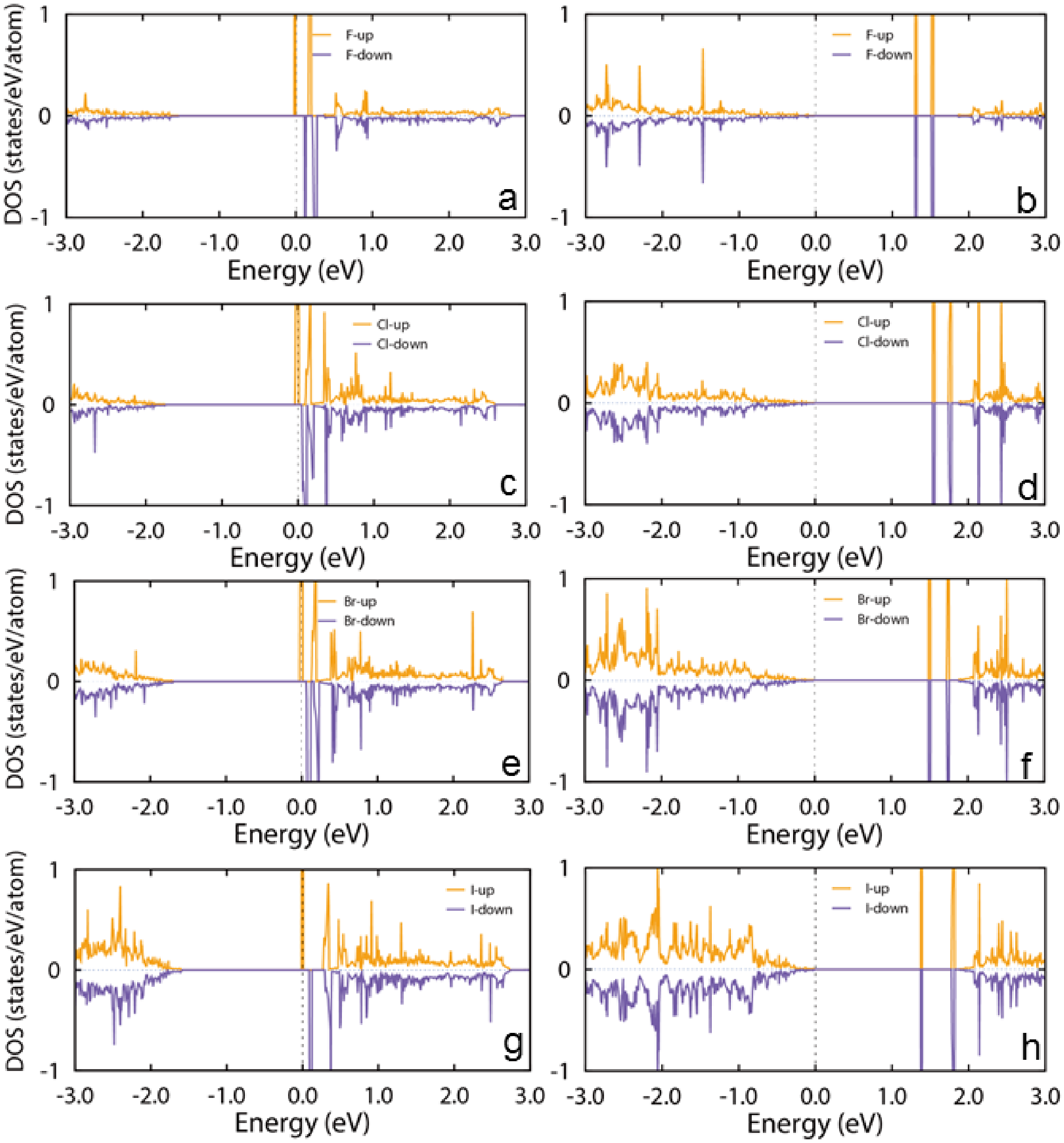

Figure S2. DOS of the substitutionally doped monolayer MoS_2 with the group-VII elements. The spin up and spin down states are shown as orange and purple solid lines, respectively. The left column is the DOS of (a) F-doped (c) Cl-doped (e) Br-doped (g) I-doped monolayer MoS_2 at neutral charge state (q=0), and the right column is the DOS of (b) F-doped (d) Cl-doped (f) Br-doped (h) I-doped monolayer MoS_2 at positively charged state (q= +1).

Charge correction method and charge transition level

In the main text, we defined the formation energies of dopant impurity at different charge states as following.

$$E_f^q = E_{tot}^{defect}(q) - E_{tot}^{pristine} - \sum N_i\mu_i + q(\varepsilon_v + \varepsilon_F) + E_{corr}. \quad (1)$$

Chemical potentials (μ_i) are determined by the total energies of the reference systems. For S and solid phase dopants such as As and Sb, the energies of bulk systems are used. For gaseous dopants such as N, the energies of gas molecules are used. For Mo, the chemical potential is determined by the difference between the total energy of monolayer MoS_2 and the chemical potential of S. The E_F ranges from 0 eV (VBM) to the band gap size 1.79 eV (CBM) in these calculations.

The charge correction energy, E_{corr}, is the correction to the total energy defined as $E_{corr} = (E_M^{iso} - E_M^{super}) + q\Delta V$, which consists of two parts; the first part is the finite-size effect that results from employing the supercell geometry, which causes the spurious interaction between images. The second part is the level alignment-like term ($q\Delta V$) due to the introduction of neutral dopant impurity and the electric potential difference between DFT calculation and the model system (explained below) when charged.

By analyzing the charge density difference between q= -1 and q=0 states of N-doped monolayer MoS_2, we find that the additional negative charge for q = -1 state is localized on the N site (see Figure S3). The plane-integrated charge density difference along x-axis (Figure S3b) and along z-axis (Figure S3c) show that the additional charge is localized at the position of N and can be modeled as a 3-dimensional single Gaussian charge centered at the dopant site. This is similar to the charge correction method implemented in our previous study for intrinsic defects such as S vacancy.[1]

Now, the charge correction can be explicitly expressed in the following form:

$$E_{corr} = E_f^q - E_{form}(\alpha) = -(\textstyle\sum_{i=1}^{5} s_i/\alpha^i + t_3/\alpha^3), \quad (2)$$

$$\text{and } E_{form}(\alpha) = E_f^q + \textstyle\sum_{i=1}^{5} s_i/\alpha^i + t_3/\alpha^3. \quad (3)$$

Here, α is the size of the supercell, fitting parameters t_3= -6.84, s_1 = -3.85, s_2 = 54.11, s_3 = -404.62, s_4 = 1401.12, and s_5 = -1851.12. Specifically, α= 6 for a 6x6 supercell and the charge correction energy calculated by plugging in α= 6 to the above expression gives 0.20 eV.

The calculated formation energy of N impurity at different charge states are shown in Fig. S4 as a function of Fermi level. The charge transition level is defined as the Fermi level at the crossing point of the two lines with different slops.

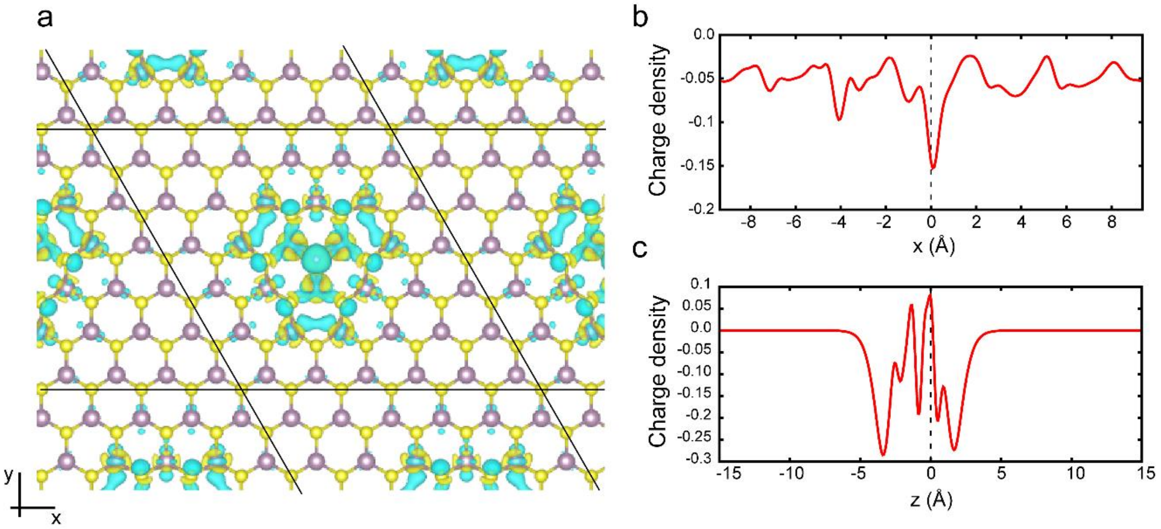

Figure S3 (a) The charge density difference of N-doped monolayer MoS_2 at charge state of q= -1 and q=0[2]. Plane-integrate charge density difference profile (b) along x direction (in-plane) and (c) along z direction (perpendicular to the plane). The position of N dopant is (0,0,0).

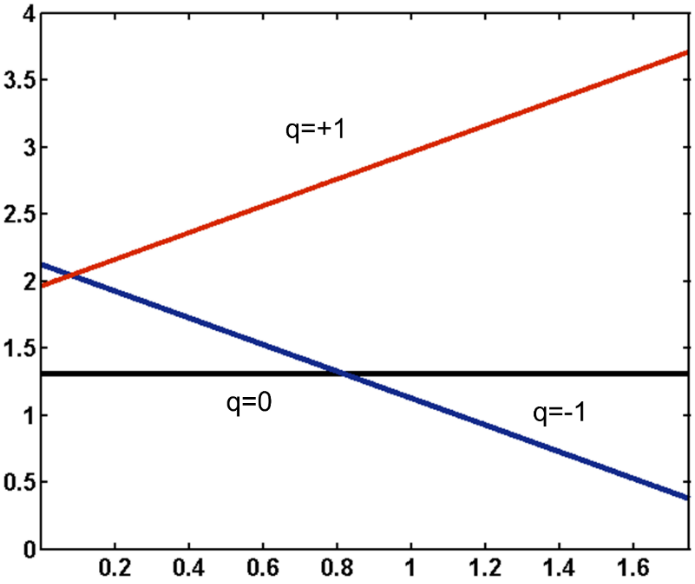

Figure S4. The formation energies of N dopant impurity with various charge states in a 6x6 supercell of monolayer MoS_2 as a function of Fermi level. The charge correction is included for each charge state, q= +1 (red) and q= -1 (blue).

Charge transition	(0/-1)				(0/+1)			
Dopant	N	P	As	Sb	F	Cl	Br	I
CTL value (from VBM)	0.865	0.532	0.468	0.738				
CTL value (from CBM)					-1.226	-0.967	-0.998	-1.104

Table S1. CTL values of Nitrogen group (N, P, As, Sb) and Halogen group (F, Cl, Br, I) dopants at S site of monolayer MoS_2.

Reference

1. Noh, J. Y., Kim, H. & Kim, Y. S. Stability and electronic structures of native defects in single-layer MoS2. *Phys. Rev. B - Condens. Matter Mater. Phys.* **89**, 1–12 (2014).
2. Momma, K. & Izumi, F. {\it VESTA3} for three-dimensional visualization of crystal, volumetric and morphology data. *J. Appl. Crystallogr.* **44**, 1272–1276 (2011).
